# The High Resolution Powder Diffraction Beam Line at ESRF

**DOI:** 10.6028/jres.109.010

**Published:** 2004-02-01

**Authors:** A. N. Fitch

**Affiliations:** ESRF, BP220, F-38043 Grenoble Cedex, France

**Keywords:** anomalous scattering, grazing incidence, microstructure, powder crystallography, residual strain, Rietveld refinement, structural solution, synchrotron radiation, x-ray diffraction

## Abstract

The optical design and performance of the high-resolution powder diffraction beam line BM16 at ESRF are discussed and illustrated. Some recent studies carried out on BM16 are described, including crystal structure solution and refinement, anomalous scattering, *in situ* measurements, residual strain in engineering components, investigation of microstructure, and grazing-incidence diffraction from surface layers. The beam line is built on a bending magnet, and operates in the energy range from 5 keV to 40 keV. After the move to an undulator source in 2002, it will benefit from an extented energy range up to 60 keV and increased flux and resolution. It is anticipated that enhancements to the data quality will be achieved, leading to the solution of larger crystal structures, and improvements in the accuracy of refined structures. The systematic exploitation of anisotropic thermal expansion will help reduce the effects of peak overlap in the analysis of powder diffraction data.

## 1. Introduction

**Fig. 1:**
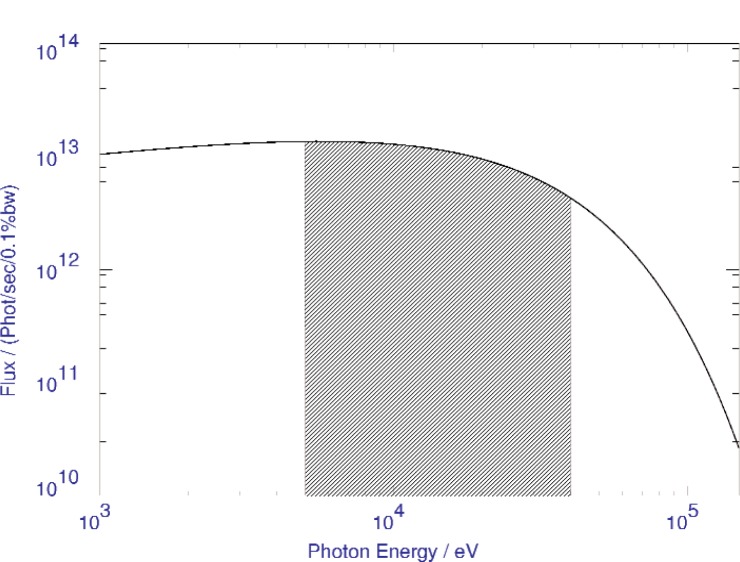
Spectrum of an ESRF bending magnet per horizontal mrad at 100 mA ring current. The shaded section shows the energy range 5 keV to 40 keV selected for BM16.

At ESRF it is possible to carry out high quality powder diffraction measurements on various beam lines, with facilities for very high temperature and pressure, anomalous scattering, rapid time-resolved measurements, etc. Two instruments in particular are dedicated to high-resolution powder-diffraction studies, the ESRF beam line BM16 and the Swiss-Norwegian beam line BM01B. BM16 has been operating with users since May 1996, and SNBL for a few months longer. Both lines are built on bending magnets and hence have the same source characteristics, Fig 1.

**Fig. 2:**
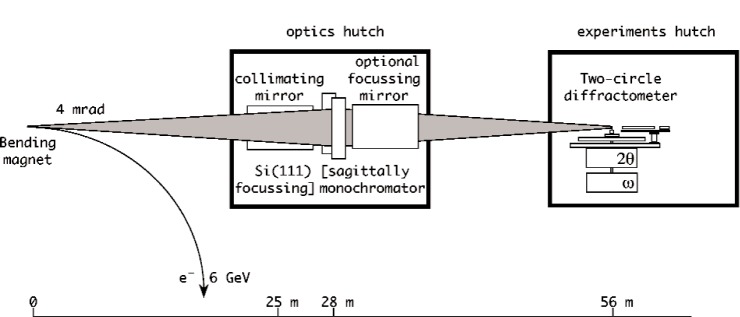
Schematic view from above of the idealised optical arrangement on BM16.

BM16 is designed to operate in the energy range 5 keV to 40 keV, (2.48 Å to 0.31 Å wavelength). The idealized optical arrangement, viewed from above, is illustrated in Fig. 2. In principle, the beam line can accept 4 mrad of white x-ray radiation in the horizontal plane from the bending magnet. This is incident on a curved mirror, set at grazing incidence, which collimates the beam in the vertical sense. After the mirror, the residual vertical divergence is around 12 µrad FWHM. It is this high degree of vertical collimation that is responsible for the high angular (2*θ*) and energy resolution of the beam line. After the mirror the beam is monochromated by a water-cooled double-crystal monochromator using Si 111 reflections. Bending the second crystal sagittally gives the option to focus the beam horizontally onto the sample.

**Fig. 3:**
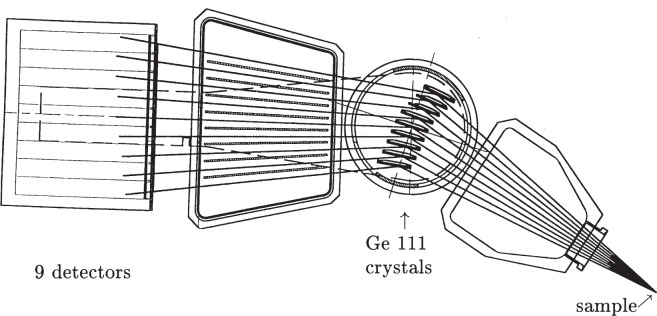
The nine-channel Ge 111 multianalyzer stage [2].

A second mirror also gives the option to focus the beam vertically onto the sample. Both horizontal [1] and vertical focusing lead to a loss of angular resolution via peak broadening, for they increase the vertical divergence of the beam incident on the sample. Operation of the beam line is nowadays, therefore, in the simplest optical configuration without additional focusing, i.e., the near-parallel beam reflected from the first mirror is monochromated and passes directly to the sample. The incident beam size is typically 5 mm × 1 mm. The diffractometer is mechanically robust, accurate (±1″) and precise (sensitive to movements of 5 × 10^–5^ °). It can accept spinning capillary or flat-plate specimens. A bank of nine scintillation detectors is scanned vertically to measure the diffracted intensity as a function of 2*θ*. The detectors are ≈2 ° apart, and each is preceded by a Ge 111 analyzer crystal. The nine Ge crystals are mounted on a single rotation stage, so only a single adjustment needs to be made when changing the wavelength. The multianalyzer stage [2] is illustrated in Fig. 3. Having nine crystals operating in parallel increases the efficiency of detecting the diffracted radiation, and for dynamic measurements means that the arm need be scanned by no more than about 2.2° to cover an angular range of 18°.

SNBL has a simpler optical arrangement for its powder diffractometer, with a water-cooled channel-cut Si 111 monochromator and no mirrors. The diffractometer is very similar to that on BM16, as the two were built in a joint project. On SNBL there are four Si 111 analyzer crystals, mounted ≈1° apart.

**Fig. 4:**
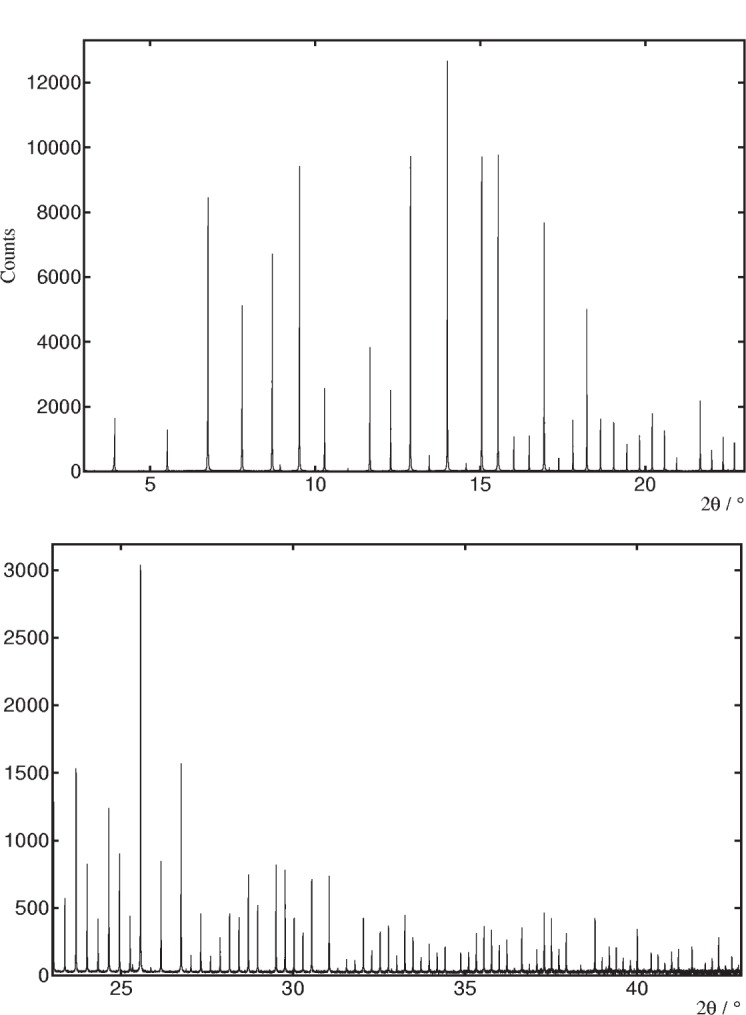
Diffraction pattern of Na_2_Al_2_Ca_3_ measured on BM16 at a wavelength of 0.48776 Å (25.42 keV).

**Fig. 5:**
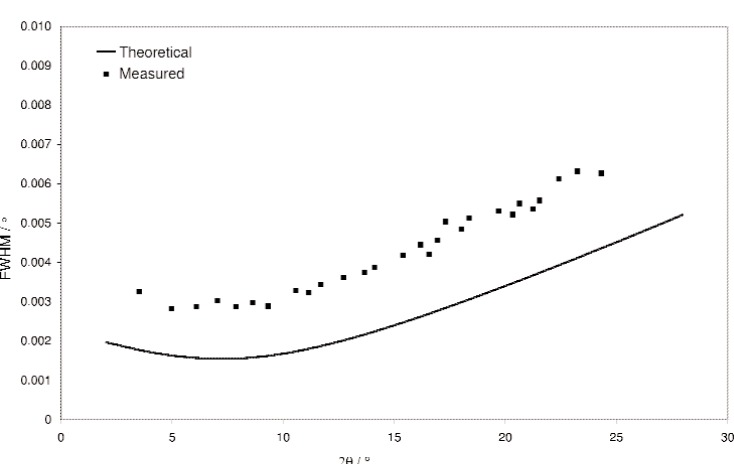
Theoretical FWHM of diffraction peaks for BM16 and those obtained from a sample of Na_2_Ca_3_Al_2_F_14_ at 28 keV ignoring the broadening due to axial divergence. See Ref. [3] for further details.

**Fig. 6:**
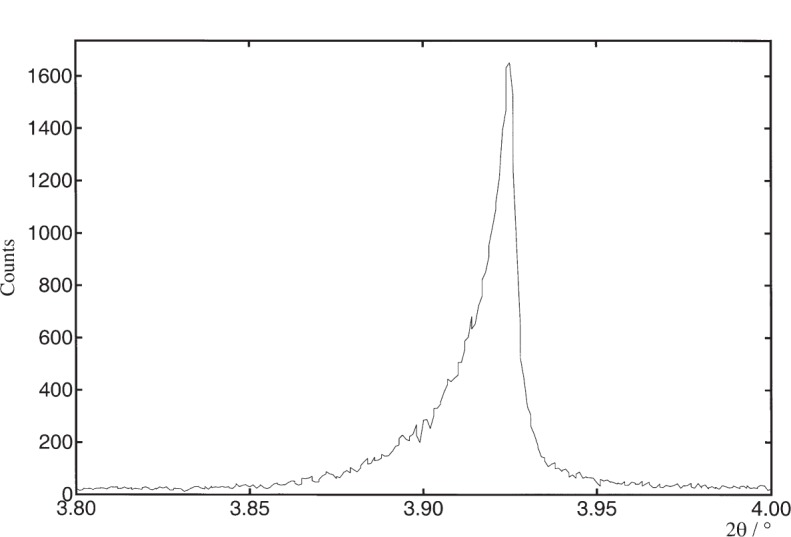
Asymmetry at low angle with Na_2_Ca_3_Al_2_F_14_.

The use of analyzer crystals means the positions of diffraction peaks are immune to aberrations that affect conventional arrangements with a scanning slit or a position-sensitive detector. Coupled with the excellent mechanical integrity of the diffractometer, not only are the peaks very narrow, with a nominal instrumental contribution to the FWHM of around 0.003° 2*θ*[3], Figs. 4 and 5, but also their positions are accurate and reproducible to a few tenths of a millidegree. Narrow peaks and accuracy in the peak positions are essential for high quality powder diffraction measurements. The analyzer crystals also offer advantages for flat-plate and grazing-incidence diffraction, and reflectivity for there is no need to be in the *θ*/2*θ* condition for optimum resolution. For crystallographic studies, the use of a spinning capillary essentially eliminates preferred-orientation effects for all but the most anisotropic and grainy of samples. With the energy range available, the energy and capillary diameter can be selected to minimize absorption even for samples containing heavily absorbing elements. Hence the diffracted intensities are also very accurate. However, working at wavelengths routinely in the range below 1 Å means that peaks are found at low-diffraction angles, where they can be significantly broadened by the asymmetry resulting from axial divergence, Fig. 6. To a good approximation, the peaks can be fitted using the function of Finger et al. [4,5].

One of the major general strengths of powder diffraction is the ability to carry out measurements under a wide range of conditions. Ancillary equipment available for experiments on BM16 includes a liquid-helium-cooled cryostat, for spinning capillary and flat-plate specimens down to 4.15 K, and below with pumping; a cold-nitrogen-gas blower for capillary specimens in the range 80 K to 373 K; a hot-air blower for capillary specimens up to 950 °C; a high-temperature oven for spinning flat-plate specimens up to 1600 °C in vacuum, and 1200 °C in an oxidizing atmosphere; a capillary cell for condensing volatile samples *in situ*, or for treatment of samples in different atmospheres or under vacuum; translation stages for mapping residual strain in engineering components.

## 2. Scientific Program

Science on BM16 can be broadly classified into categories such as

- Powder crystallography, solving and refining of crystal structures,
- Anamalous-scattering studies, tuning to absorption edges to help distinguish neighboring elements, oxidation states, or to help characterize disordered structures,
- Dynamic or *in situ* measurements, following changes with temperature or time, or in electrochemical cells, etc.,
- Measurement of residual strain in bulk materials and surfaces,
- Characterization of microstructure, from the analysis of peak shapes,
- Diffraction at grazing incidence from surfaces, and reflectivity.

The categories are not mutually exclusive, e.g., resonant scattering is usually part of a more general investigation of the crystallographic structure of a material, and reflectivity can be carried out at the absorption edges of the components of multilayers, or the kinetics of the evolution of microstructure can be investigated, etc.

### 2.1 Powder Crystallography

As mentioned above, the combination of narrow peaks, accurate peak positions and intensities has advantages for crystallographic measurements. Powder diffraction patterns are routinely measured using x-rays with a wavelength in the range 0.3 Å tο 0.9 Å, with capillary diameters of 0.3 mm to 1.5 mm, to keep µr < 1.5. The resolution and angular accuracy are especially important for indexing of powder patterns, the identification of possible space groups, and the extraction of intensities by a Le Bail or Pawley-type fit. With high incident flux, diffraction patterns suitable for structure solution can be measured in a number of hours. For very weakly scattering materials, more time is usually necessary. Some examples of structures solved or refined exploiting the attributes of BM16 include a number of pharmaceutical organic compounds by David et al. [6], making use of their own simulated annealing program to define the positions, orientations and conformations of the molecules in the structure, and fluorescein diacetate [7], C_24_H_16_O_7_, where the molecular structure and triclinic crystal structure were solved without any prior crystallographic information by direct methods. Ba_4_C_60_ [8] was refined in space group *Immm* above and below the superconducting transition, in the presence of small amounts of Ba_6_C_60_ and Ba_3_C_60_ side products. The occurrence of superconductivity in this non-cubic fulleride was rationalized in terms of hybridization between the fulleride ions and one of the two crystallo-graphically distinct Ba ions, as revealed by the close contacts. Also ErFe_4_Ge_2_ [9], which disproportionates below the Neél temperature at 44 K into two different phases, with different magnetic properties, whose relative proportions vary sharply with temperature in response to magnetostriction effects. For the material reported to be a metastable form of dimethylsulpfide dibromide, (CH_3_)_2_SBr_2_, the high angular accuracy of the data, measured on the Swiss-Norwegian beam line, allowed the diffraction pattern to be indexed as a mixture of two previously unknown phases [10]. The structures were solved by direct methods from the powder data and were found to be orthorhombic (CH_3_)_2_SBr_2.5_ and monoclinic (CH_3_)_2_SBr_4_, each containing more bromine than required for the formulation as a metastable phase of (CH_3_)_2_SBr_2_.

Despite the capacity to solve relatively complex organic structures such as fluorescein diacetate by direct methods, obtaining all the carbon and oxygen atoms in the molecule with bond distances to within about 0.1 Å of what would be expected, the accurate refinement of structures still presents considerable difficulties. For fluorescein diacetate, restraints were required to prevent unacceptable distortions to the molecule. For the simple bicyclic molecule norbornene, C_7_H_10_, which has mirror symmetry, the refinement of the molecular structure, restrained to *C_s_* symmetry in space group *P*2_1_/*c*, was in good agreement with the results of an *ab initio* molecular orbital optimization of the structure [11]. However, for the case of camphor, C_10_H_16_O, a slightly bigger molecule that has no special molecular symmetry and two molecules in the asymmetric unit, the agreement was less good [12]. As is well known, problems arise because of the limitations of powder data caused by peak overlap and, usually in the case of x ray, weak statistics at high angle, limiting the amount of crystallographic information that can be reliably extracted. When peak overlap is severe, owing to the sheer number of reflections from a complex structure, the intensities and the background are ambiguous which is a further hindrance. The description of the thermal motion of the molecule must often be restricted to no more than an overall isotropic temperature factor, which may be a poor approximation to the situation in the crystal. Thus, even with high quality powder-diffraction data, there are still obstacles to be overcome to improve the reliability of refined structures.

### 2.2 Anomalous Scattering

Resonant scattering experiments have been performed at edges spanning the energy range available on BM16. Experiments at low energy, below 8 keV, at the K edges of the early transition metals and the L edges of early lanthanides have been more difficult to perform, because of absorption by the Be windows in the beam line and by the samples themselves. Flat-plate samples are necessary at these edges, for which the multianalyzer stage is less well optimized. Only one detector can be in the *θ*/2*θ* arrangement, for which the effect of absorption on the intensity is isotropic (for a sufficient depth of powder). Hence corrections need to be made to the intensities coming from the other eight detectors. Under ambient conditions, the Mn edge at 6.5376 keV (1.8964 Å) has proved to be the practicable limit. An elegant experiment was performed by Joubert et al. [13], in which data from the Ni, Co, and Mn edges were used in conjunction with a scan away from all edges to unravel the distribution of cations over two sites in the battery-electrode material LaNi_3.55_Mn_0.4_Al_0.3_Co_0.75_. Resonant scattering studies at higher energies have also worked well, e.g., at the Ag K edge (25.5165 keV, 0.4859 Å) in Ag-Y zeolite [14] and Ag substituted ZSM-5, or at the La K edge (38.934 keV, 0.3184 Å) in La_1.2_Sr_1.8_Mn_2_O_7_, a layered magnetoresistive perovskite, to increase the sensitivity of the refinement to the distribution of La and Sr over the available sites [15]. At such energies capillary samples can be employed, with the energies chosen at and just below the edge.

### 2.3 Dynamic Measurements

Dynamic measurements have included the exploration of phase diagrams with temperature, the kinetics of chemical reactions studied *in situ* such as dehydration, or hydrothermal synthesis, or following the changes in the crystalline components in electrochemical cells during the electrochemical cycle. By working at an energy such as 31 keV, the complete battery can be put in the beam, and high-resolution powder diffraction patterns of all components measured in transmission during the charging and discharging cycles, with a time resolution of around 20 min. For example, for Belcore plastic batteries, which are about 2 mm thick, the intercalation of Li ion in and out of the Li_x_Mn_2_O_4_ electrode material, or a fluorine-substituted analogue Li_x_Mn_2_O_3.74_F_0.26_, was followed [16].

An example following physical evolution is the measurement of the kinetics of grain growth in nanocrystalline iron prepared by pulsed electrodeposition [17]. The aim was to understand the factors that influence the final grain size, which may influence properties such as coercivity and permeability in ferromagnetic materials, hardness, and corrosion resistance. The temperature was increased rapidly to the desired growth temperature, and a few diffraction peaks monitored by scanning the nine detectors over 2.5° on the time scale of a minute, (registering data every 10 ms), which was the fastest practicable. Depending on the temperature, the growth of the particles lasted between a few minutes, and several hours. From a careful analysis of the peak shapes, the temporal evolution of grain size, grain-size distribution and microstrain were followed. The intrinsically narrow instrumental contribution of BM16 to the FWHM allowed the particles’ growth to be followed to a grain size of about 500 nm, whereas for a laboratory instrument the limit is 100 nm. The energy used (25 keV) allowed the bulk of the sample to be analyzed, rather than just the surface. However to improve statistical precision, it was necessary to add together the data in blocks of 5 min elapsed time. This is an experiment that would clearly benefit from more incident intensity. Nevertheless, two different regimes of growth were observed, depending on temperature, and the quality of the data allowed a critical evaluation of different kinetic models of grain growth. In a similar experiment [18] when nanocrystalline Fe was prepared using the technique of mechanical attrition, measurements of grain growth revealed a linear dependence of the grain size on annealing time, contradicting studies in coarser-grained materials, which had a parabolic (or power-law) dependence. Beyond about 150 nm, a smooth transition from linear to nonlinear growth kinetics occurred, suggesting that the rate-controlling mechanism for grain growth depends on the grain size. The linear-stage growth rate was in quantitative agreement with a model in which the redistribution of excess volume localized in the boundary cores controls the boundary migration.

### 2.4 Residual Strain

**Fig. 7:**
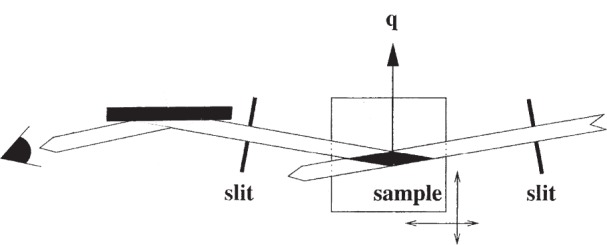
The entrance and exit slits, and the analyzer crystal define a gauge volume. The position of a diffraction peak is measured from different parts of the sample, thus mapping the strain distribution.

The energy range available on BM16 is well suited to measurements of residual strain in components made in light alloys, (Al- or Ti-based), linked for example to aerospace applications [19]. Fine entrance and exit slits, 50 µm to 100 µm high, and the analyzer crystal define a gauge volume. The component is then translated around and the position of a diffraction peak measured for different parts of the sample, Fig. 7, thus mapping the residual strain. Work has been carried out around welds [20,21], e.g., produced by the novel friction-stir-welding process, or in surfaces treated by peening [19,22] to induce a protective layer under a compressive stress, so that damage closes up rather than propagates. The depth profile of the compressive stress and the appearance of the deeper balancing tensile stress can be probed down to several tenths of a millimeter as a function of the peening process. BM16 has significant advantages. Firstly the hard energies allow penetration through substantial pieces of material, e.g., 15 mm of Al alloy, or 3 mm of Ti alloy. Secondly, by using the analyzer crystal the peak positions are immune to geometrically induced surface aberrations, for the real angle of the diffracted radiation is defined by the analyzer, rather than inferred from the position of a slit. This is crucial when moving the surface of the specimen through the gauge volume. The analyzer crystal also leads to narrow peaks and very small changes in peak position are readily observed. Hence the experiment has enhanced accuracy, sensitivity and precision.

### 2.5 Characterization of Microstructure

Owing to the very small instrumental contribution to the peak widths, sample effects dominate the peak shapes for most samples. This is a limiting factor for high-resolution studies for solving and refining crystal structures, but is useful in investigating microstructural aspects of a material. Examples include the study of the kinetics of grain growth in nanocrystalline Fe, given above [17,18], the characterization of stacking faults in the magnetoresistive layered perovskite La_1.2_Sr_1.8_Mn_2_O_7_ [15], and the characterisation of size and dislocation-induced strain broadening in gold films, 300 Å to 1900 Å thick, by symmetric *θ*/2*θ* scanning and asymmetric transmission measurements using 0.3507 Å (35 keV) radiation [23].

A novel study [24] investigated ancient Egyptian cosmetic powders. The composition, in terms of the mixture of phases present, had already been determined by Rietveld refinement from the high-resolution powder diffraction patterns [25]. The microstructure of the components can give clues as to the elaboration process via crushing or sieving, etc. The peak profiles of galena (PbS), a major ingredient in the make up, were compared with those from samples of geological galena (crushed and sieved, and also finely crushed), and synthetic PbS. The archeological specimens and the crushed specimens show distinct anisotropy in the microstructure. The analysis confirms that galena was more or less finely ground by the Egyptians and sorted as a function of size, to obtain either a black matt powder, or grey powders with metallic overtones. Another component of some cosmetics, laurionite (PbOHCl), had similar peak breadths and SEM morphology to synthetic laurionite powders. This reinforces the earlier conclusion [25], based on the fact that the natural abundance is very rare and by recipes found in ancient Egyptian texts, that the laurionite had been prepared synthetically, as was phosgenite, (Pb_2_Cl_2_CO_3_), another ingredient found in some powders.

### 2.6 Surfaces and Reflectivity

Measurements at grazing incidence and reflectivity have been carried out to quantify the surface roughness in systems of spin valves [26]. At wavelengths of 1.381 Å or 1.48 Å, specular (*θ*/2*θ*) reflection, longitudinal diffuse measurements (*θ* offset by –0.1° from 2*θ*), and transverse diffuse scans (fixed 2*θ*, scan sample), were performed. Large differences were seen in the roughness of SiO_x_ substrate produced by thermal oxidation or by low-pressure chemical vapor deposition and this was propagated through to the spin valves themselves, influencing their giant magnetoresistive properties.

Bragg peaks from ground-and-polished alumina surfaces were investigated with 8 keV x rays at incidence angles from 0.25° to 20°, corresponding to penetration depths from 0.3 µm to 25 µm [27]. Damage extends below the surface, and the FWHM were found to decrease exponentially with depth from maximum values of 0.58° when ceria polish was used, or 0.178° with diamond polish. The peak positions were however independent of depth, once correction had been made for the effect of refraction of the incident radiation.

## 3. Future Developments

Since the construction of the ESRF there have been significant developments in the performance of insertion devices [28], with small-gap undulators and fine tuning of the magnets to allow very high brilliance x rays and high-energy operation with high-order harmonics. To exploit these developments it has been decided to transfer the diffractometer of BM16 from its current position on a bending magnet to a new beam line with an insertion device, ID31. The transfer will take place in March 2002.

The new beam line will have three undulators with a minimum gap of 11 mm, allowing the energy range from 5 keV to 60 keV (0.21 Å) to be covered without interruption. No optical elements are planned other than a liquid-nitrogen-cooled double-crystal monochromator with a choice of Si 111 or Si 311 crystals. The undulator beam will be highly collimated in the horizontal and the vertical directions, and will result in a significant increase in the flux incident on the sample compared with BM16.

It is intended to exploit the increased flux to improve the quality of the data from the instrument. By decreasing the axial dimensions of the beam on the sample from the current 4 mm to 5 mm to 1 mm to 2 mm, and the receiving slit from 10 mm to say 1 mm to 2 mm, the marked asymmetry at low angle can be reduced, along with the associated peak broadening and peak shifts. As well as being easier to fit, this should enhance the resolution and may be important in the indexing of patterns with large unit cells. The overall instrumental resolution will also be improved by the use of Si 111 analyzer crystals in place of the Ge 111 crystals. Better statistical quality in the data, particularly at high angle is also expected, which should lead to improvements in the size and accuracy of the structures that can be solved and refined. The generally poorer statistical quality of the high angle data is a factor that limits current performance. Counting schemes to compensate for this have been proposed, which suggest that the majority of the acquisition time should be spent in the high-angle region [29].

Higher flux will also mean that data can be collected more quickly. Weakly scattering or very tiny amounts of material can be investigated more readily, and for systems evolving with time or temperature, more points can be investigated. The structural parameters can be fitted to a series of patterns, maybe restrained to follow a theoretical or empirical model, thus revealing more about the underlying physics or chemistry of the structural evolution. For static structures, data can be collected systematically at a number of temperatures, allowing any anisotropic thermal expansion of the solid to be exploited to help disentangle the intensities of overlapping peaks, particularly at high diffraction angle. Peaks closely overlapping at one temperature may be better resolved at another. The ambiguities and correlations inherent in powder data will therefore be reduced, and this should improve the accuracy of structures refined, and the complexity of structures that can be solved. The utility of such an approach in the solution of a molecular crystal structure has already been demonstrated [29].

An increase in the energy range to 60 keV will give access to the K edges for most of the rare-earth elements, which can provide complementary information to the L-edge experiments currently possible. The K edge has the advantage that the experiment can be carried out in capillary geometry because the absorption is very much less than at the softer L edges, although the anomalous scattering factors are smaller. For strain measurements, the absorption of Al falls by a factor 2 as compared to 40 keV, and for Ti and heavier metals by a factor near 2.8. Hence the penetration into light alloys will be increased allowing bulkier components to be investigated. ID31 will be operational in mid 2002.
